# Risk assessment for cardiovascular adverse drug events in the ICU: Case study on COVID-19 patients

**DOI:** 10.1371/journal.pone.0345280

**Published:** 2026-03-24

**Authors:** Natalia L. Freitas, Raiane Diniz Oliveira, Ana Katarina da Silva Santos, Marcilio Cunha-Filho, Patricia Medeiros-Souza

**Affiliations:** 1 School of Health Sciences, University of Brasilia (UnB), Brasília, DF, Brazil; 2 Department of Health of Distrito Federal (SES-DF), Brasília, DF, Brazil; 3 Laboratory of Food, Drugs, and Cosmetics (LTMAC), University of Brasilia, Brasília, DF, Brazil; Deccan School of Pharmacy, INDIA

## Abstract

The COVID-19 outbreak quickly became a pandemic. In Brazil, more than seven million cases were recorded in 2020. Most patients affected by the disease were admitted to intensive care units (ICU), requiring qualitative polypharmacy, increasing the risk of serious adverse drug reactions (ADE), especially cardiovascular. This study aimed to identify cardiovascular ADE in the ICU of a reference hospital for the treatment of COVID-19 in Brasília, Brazil. The Tisdale score was determined by analyzing 141 medical records of patients with COVID-19 admitted in 2020. Furthermore, the MedUTI software was used to simulate hypothetical drug substitutions to reduce cardiovascular risk. The result showed that after simulating the hypothetical intervention of prescriptions, a 53% decrease in high-risk patients was observed (from 96.0% to 43.0%). In this optimized prescription condition, 56% of them began to show a Tisdale score of medium or low risk. In addition, a decrease from 67 to 51 of medications with serious cardiovascular ADE was noted, i.e., QT interval prolongation (from 37 to 30), Torsades de Pointes (from 21 to 15), and SS (from 9 to 6). The COVID-19 medical emergency has highlighted the need for rapid medication management in ICU patients. Thus, the use of technologies to support healthcare professionals’ work can be decisive for patient survival, especially in ICU overcrowding scenarios.

## 1. Introduction

In 2020, Brazil had more than 7 million cases of COVID-19 and almost 200 thousand deaths, with incidence and mortality rates of 3,653/100 thousand inhabitants and 93/100 thousand inhabitants, respectively. During this period, the Federal District accounted for 251,701 cases, and 4,259 deaths had already been recorded, with an incidence rate of 8,348/100 thousand inhabitants and a mortality rate of 141/100 thousand inhabitants [1]. The unprecedented pandemic caused by SARS-CoV-2, officially declared by the WHO in March 2020 [2,3], caused the death of millions of people worldwide. So far, there is no effective treatment for the infection.

Patients with severe COVID-19 were invariably admitted to the intensive care unit (ICU). Even without effective drug treatment against the virus, such patients presented a very complex clinical picture requiring the simultaneous administration of several drugs (quantitative polypharmacy) [4]. In addition, pre-existing conditions required additional drug intervention, and the incidence of self-medication was relatively high, considering the collapse of the health system in many countries [5].

In clinical scenarios such as these, there is an increased risk of serious adverse drug events (ADE) and ADE synergism. Serious ADE can lead to death or hospitalization, disability, and congenital anomalies [6]. Medical reports from the period indicate that cardiovascular reactions were particularly relevant for these patients [7–10]. Additionally, the SARS-CoV-2 virus induces significant cardiac impairment during infection, making ADE at the cardiovascular level even more critical [7,11,12].

The frequent serious cardiovascular ADE in ICU patients diagnosed with COVID-19 mainly involves QT interval prolongation [13–16]. This condition is characterized by the prolongation of the interval observed on the echocardiogram. An aggravated form of this condition is the occurrence of Torsades de pointes, characterized by ventricular tachycardias that increase the risk of sudden cardiac death [17]. Torsades de pointes is a polymorphic form with a QT-interval-prolongation morphology, initiated after early depolarization [18]. Finally, another serious ADE to be considered in the cardiovascular context is serotonin syndrome (SS), which is related to excess synaptic serotonin in the central nervous system, producing neuromuscular excitation, autonomic effects, and altered mental status [19].

Despite the critical aspect of cardiovascular ADE in the COVID-19 pandemic scenario, a comprehensive study on rational medication management with a focus on reducing cardiovascular risks for ICU patients has never been conducted. In this context, finding technological assessment and support tools to improve the current medication treatment protocols in the ICU can greatly benefit health professionals and patients, especially in ICU overcrowding and health emergencies.

Thus, this study aimed to identify cardiovascular ADE in the ICU of the COVID-19 reference hospital in Brasília by analyzing medical records of patients with COVID-19 admitted in 2020 (pre-vaccination period). To this end, the cardiovascular risk associated with the patient’s prescriptions was determined using the Tisdale score and the MedUTI software. Based on this assessment, a hypothetical drug substitution was proposed to reduce the cardiovascular risks of these prescriptions.

## 2. Materials and methods

### 2.1 Delimitation of the population

The medical records of adults with COVID-19 hospitalized in the ICU of the reference hospital for COVID-19 in Brasília/Brazil, from March to December 2020, were compiled. The selected patients were over 18 years old, of both sexes, and diagnosed with COVID-19 by RT-PCR laboratory test, rapid test, or symptomatological diagnosis.

From September 1–29, 2023, 141 medical records were accessed retrospectively. Patients were identified by medical record code to avoid their identification during data collection. Patients who lacked information about previous hospitalizations in another hospital or without electronic medical records were excluded. Thus, 106 medical records were eligible for the study. The study covered access to patients’ demographic information, their comorbidities and health conditions, the outcome after hospitalization, and, especially, the daily use of medicines during hospitalization. Moreover, comorbidities and health conditions were classified according to the Anatomical Therapeutic Chemical system [20].

The research was approved by the Ethics Committee in Human Research of the Faculty of Health Sciences (University of Brasilia, Brazil) with approval number 5,644,356 and by the Ethics Committee in Research of the Foundation for Teaching and Research in Health Sciences (Federal District, Brazil) with approval number 5,723,352. The study protocol followed the guidelines and regulatory standards for human research [21]. All surviving patients and relatives, in the case of those who died, were contacted by phone and consented to participate in the research through the Informed Consent Form. The contact was documented using a printout of the cell phone screen.

### 2.2 Study methodology

An analytical observational study was conducted with a retrospective longitudinal design. The primary objective was to evaluate the cardiovascular risk of prescriptions, in particular, in assessing the synergism of serious ADE related to QT interval prolongation. To this end, the cardiovascular risk of the prescriptions of COVID-19 patients examined was determined based on a score proposed and validated by Tisdale, 2023, with adaptations.

Additionally, the MedUTI software [22] was used to identify serious cardiovascular ADE and the synergism of serious ADE, as well as a reference tool for medication replacement. The software database is composed of cross-referencing clinical information based on evidence about serious and common pharmacodynamic interactions.

### 2.3 Cardiovascular risk determination

The Tisdale score [23] was calculated from clinical and sociodemographic variables. In short, the sociodemographic characteristics used to score cardiovascular risk included advanced age (≥68 years; 1 point) and female gender (1 point). Clinical variables scored in the model were the use of diuretics (1 point), biochemical factors such as serum potassium concentration ≤3.5 mmol/L (2 points), and QT interval ≥450 ms (2 points). In addition, drug associations were scored in cases that presented 1 drug with possible serious QT interval prolongation (3 points) and when there were 2 or more drugs that presented possible serious QT interval prolongation (an additional 3 points). The cardiovascular comorbidities were also scored, including the history of acute myocardial infarction (2 points), congestive heart failure with reduced ejection fraction (3 points), and the occurrence of sepsis (3 points). Based on the total score, prescriptions were classified as low (<6), moderate (7–10), or high risk (≥11).

The daily cardiovascular risk, as determined by the Tisdale score, was calculated exclusively from the drug associations prescribed and effectively administered during the patient’s ICU stay. Chronic-use medications and self-medication drugs used prior to hospitalization were collected solely for sociodemographic and clinical characterization of the population and were not included in the score calculation.

An analysis was performed on prescriptions corresponding to the days when these patients had two or more synergistic associations, allowing changes in cardiovascular risk throughout hospitalization to be captured. Using the MedUTI software, synergism related to QT interval prolongation was identified across six therapeutic classes (Fig 2), whose associations substantially increased the probability of severe cardiovascular ADE. More serious ADE, such as Torsades de pointes and serotonin syndrome (SS), were also screened using MedUTI.

**Fig 1:**
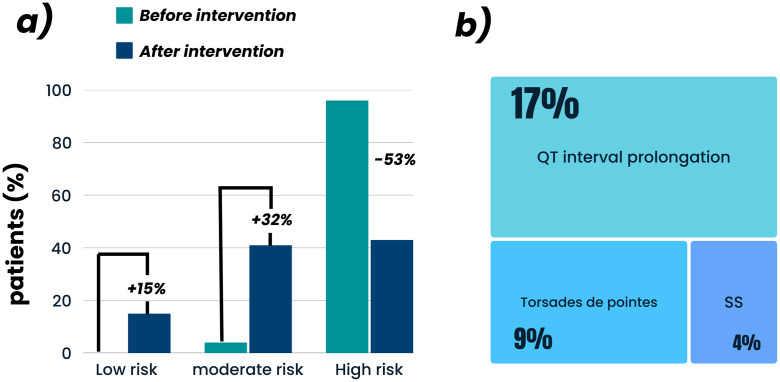
Analysis of the Tisdale score before and after the hypothetical intervention of prescriptions (a), together with the serious cardiovascular ADE in percentage (b). Serotonin syndrome (SS).

**Fig 2:**
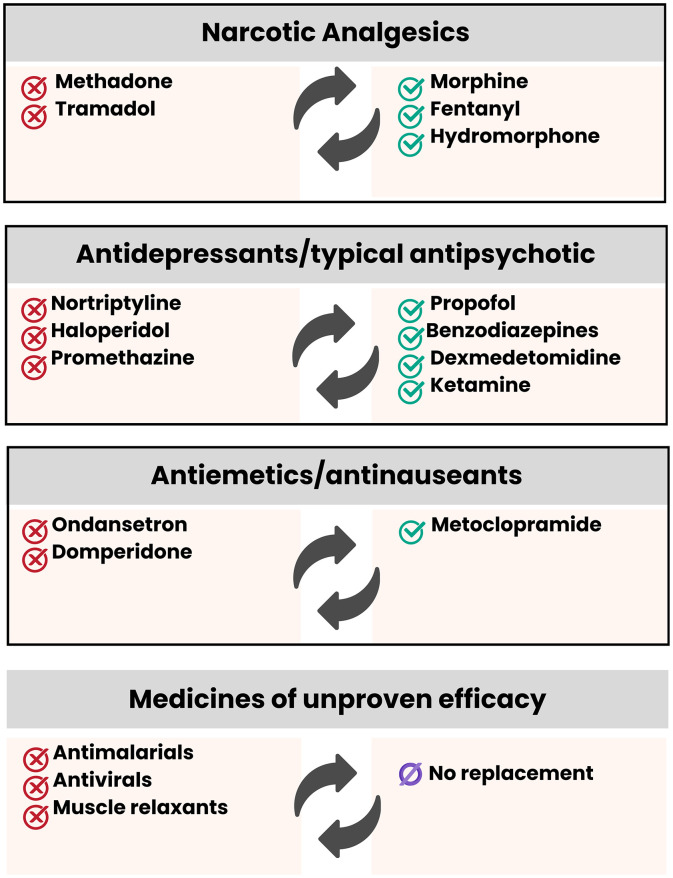
Clinical management of medications prescribed in the ICU with the potential for serious adverse reactions caused at the cardiovascular level.

The evaluation of cardiovascular risk and the identification of serious ADE were performed before and after the hypothetical clinical management simulated in this study. Drug substitution criteria were defined to reduce cumulative cardiovascular risk. These substitutions were likewise applied, ensuring that the estimated impact on cardiovascular risk reflected each patient’s individual and time-dependent clinical trajectory.

### 2.4 Statistical analysis

An analysis of the selected population of patients with COVID-19 in terms of demographic, socioeconomic, and clinical history variables was performed. For categorical variables, absolute and relative frequencies were presented, while continuous variables were summarized by mean and standard deviation. Multiple binary logistic regression was used to identify the main variables associated with patient clinical outcomes. Independent variables that showed a high correlation with the outcome or with each other were excluded due to multicollinearity problems. The significance of the regression parameters was tested using the Wald statistical test.

To compare cardiovascular risk scores before and after hypothetical drug therapy, a nonparametric paired Wilcoxon test was used, as both measurements were obtained from the same individuals. Additionally, a comparison of the Tisdale score with stratification by some variables was performed using the two-factor ANOVA test, the Wilcoxon test, and QQ plot analysis.

Then, to evaluate this difference in the presence of other covariates, maintaining the same demographic, socioeconomic, and clinical variables, the variance analysis test (ANOVA) with two factors was applied. To compare Tisdale scores within each category, the paired nonparametric Wilcoxon test was used.

Additionally, a regression model was carried out with the Tisdale score classification as the response variable, and prevalence ratios were used to measure the risk of increased Tisdale score across the different categories of each independent variable. The model employed was a generalized linear model with the Poisson family and a log link function.

The analyses were performed using R version 4.2.2 (31 December 2022). The significance level used in this study was 5%.

## 3. Results

After the exclusion criteria, 106 medical records were considered for the present study. The qualitative variables are presented by frequency (n) and percentage (%) in Table 1, including the sociodemographic and clinical data.

**Table 1:** Descriptive analysis of sociodemographic and clinical variables of patients with COVID-19 from March to December 2020.

Demographic characteristics		n	%
	Total	106	100.0
**Age**	25 to 39 years old	8	7.0
	40 to 49 years old	15	14.0
	50 to 59 years old	24	23.0
	60 years and over	59	55%
**Sex**	Male	69	65.0
**Race**	White	13	12.0
	Multiracial	27	25.0
	Unknown	66	62.0
**Comorbidity**	None	22	21.0
	One	34	32.0
	Two	22	21.0
	Three or more	28	26.0
**Health conditions**	None	13	12.0
	One	45	42.0
	Two	32	30.0
	Three or more	16	15.0
**Addiction**	Alcoholic	20	19.0
	Smoker	34	32.0
	Illicit drug user	3	3.0
**Comorbidities involving medication use by the system**			
**Cardiovascular and renal system**	Arrhythmia, acute myocardial infarction, systemic arterial hypertension, and congestive heart failure	107	–
**Metabolism disorders**	Dyslipidemia, obesity	57	–
**Endocrine system**	Diabetes mellitus	38	–
**Nervous system**	Anxiety and/or depression, Alzheimer’s or dementia, stroke	24	–
**Respiratory system**	Asthma, chronic obstructive pulmonary disease	12	–
**Immune system**	HIV-positive, cancer, drug immunosuppression	7	–
**Chronic medications and self-medication**			
**Chronic medication before hospitalization**	Yes	69	65.0
**Chronic medications that cause QT interval prolongation**	Propranolol, clonidine, promethazine, amiodarone, amitriptyline, sertraline, escitalopram, nortriptyline, haloperidol, risperidone, and tacrolimus	15	14.0
**Self-medication before hospitalization**	Yes	21	20.0
**Self-medication causing QT interval prolongation**	Azithromycin, chloroquine, hydroxychloroquine, and moxifloxacin	18	17.0
**Hospitalization information**			
**Length of stay in the hospital’s ICU**	<7 days	18	17.0
	7 to 13 days	13	12.0
	14 to 20 days	29	27.0
	21 to 27 days	18	17.0
	28 days or more	28	26.0
**Outcome**	Discharge due to death	78	74.0
	Discharge with sequelae	10	8.0
	Transfer to another hospital	10	9.0

Male patients were prevalent (n = 69; 65.0%), most over 60 years old (n = 59; 55.0%). Most patients had at least one health condition (n = 93; 88.0%), presenting, in some cases, three or more (n = 16; 15.0%). Considering patients with dependence, smoking was the most prevalent (n = 34; 32.0%), followed by alcoholism (n = 20; 19.0%).

Furthermore, most patients had some comorbidity (n = 84; 79.0%), with a high prevalence of patients with two or more comorbidities (n = 50; 47.0%). In the case of comorbidities involving the cardiovascular and renal systems, the most prevalent disease was systemic arterial hypertension (SAH) (n = 61, 58.0%), followed by acute myocardial infarction (AMI) (n = 8; 8.0%), arrhythmia (n = 5; 5.0%), and congestive heart failure (CHF) (n = 2; 2.0%). In the endocrine system, obesity was the most prevalent condition (n = 61; 58.0%), followed by Diabetes mellitus (n = 38, 36.0%).

Among the patients studied, 69 (65.0%) made chronic use of medications, and 14% (n = 15) used at least 1 medication that causes serious ADE of QT interval prolongation, i.e., propranolol, clonidine, promethazine, amiodarone, amitriptyline, sertraline, escitalopram, nortriptyline, haloperidol, risperidone, and tacrolimus.

Regarding self-medication, 21 patients (20.0%) used at least 1 medication to treat COVID-19 on their own, often anti-infective (50%, n = 27) and antiparasitic agents (22%, n = 12). Within this group, 17% (n = 18) used at least 1 medication that causes serious ADE of QT interval prolongation, including azithromycin, chloroquine, hydroxychloroquine, and moxifloxacin.

Patients were hospitalized for an average of 29 days, with 21 of those days spent in the ICU and a higher prevalence of discharge due to death (n = 78; 74%). The evaluation of variables associated with the patient’s clinical outcome in the multivariate model indicated that for each additional year of age or day of hospitalization, the chance of the patient dying from COVID-19 increased by 1.093 (p = 0.003) and 1.148 (p = 0.002), respectively.

Finally, considering all medications used by patients in this study, including those prescribed during hospitalization, self-medication, and chronic use, 21 medications (9.0%) presented serious ADE of renal failure, 14 (6.0%) were nephrotoxic, 7 (3.0%) caused hearing loss, and 5 (2.0%) were ototoxic. The sequelae of COVID-19 identified in patients were respiratory (n = 4; 4.0%), hearing loss (n = 3; 3.0%), neurological (n = 3; 3.0%), motor (n = 3; 3.0%), hypertension (n = 2; 2.0%), visual loss (n = 1; 1.0%), and renal failure (n = 1; 1.0%). Some patients presented more than one sequelae.

From the analysis of these patients’ prescriptions, a cardiovascular ADE assessment was performed before and after the hypothetical intervention described in Fig 1a to capture cumulative exposure burden throughout the ICU stay. Among the items analyzed by the Tisdale score, it was possible to observe that before the intervention simulation, no prescription presented a low risk of developing QT interval prolongation, 4 prescriptions (4.0%) presented a moderate risk, and an impressive 102 prescriptions (96.0%) were classified as high risk.

Moreover, from the 6,149 medications prescribed during the hospitalization of all patients involving 220 different drugs, 67 (30.0%) cause serious cardiovascular ADE, in which 37 (17.0%) of QT interval prolongation, 21 (9.0%) Torsades de pointes, and 9 (4.0%) SS (Fig 1b).

Based on the MedUTI software, the hypothetical interventions to be performed and the therapeutic classes in which adjustments were made, including changes or withdrawals of medications, are outlined in Fig 2 and detailed below.

(i) Replacement of the opioid narcotic analgesics methadone and tramadol with morphine, fentanyl, and hydromorphone [24–26];
(ii) Replacement of tricyclic antidepressants (nortriptyline) and antipsychotics associated with antihistamines (haloperidol and promethazine) with sedative drugs (propofol, benzodiazepines, dexmedetomidine, and ketamine) [27];
(iii) Replacement of antiemetic/antinauseant drugs (ondansetron and domperidone) with dopaminergic antagonist drugs with antiemetic and peristalsis-stimulating action (metoclopramide) [28];
(iv) Withdrawal of antimalarial drugs (chloroquine, hydroxychloroquine), which were considered possible treatment alternatives but proved ineffective [29,30];
(v) Withdrawal of antiviral anti-HIV drugs (lopinavir + ritonavir), which were considered possible treatment alternatives but proved ineffective [29,30];
(vi) Withdrawal of drugs from the pharmacological class of muscle relaxants (cyclobenzaprine), which were considered possible treatment alternatives but proved ineffective [29,30].

After simulating the hypothetical intervention of prescriptions, a 53% decrease in high-risk patients was observed (from 96.0% to 43.0%). In this optimized prescription condition, 56% of them began to show a medium or low risk of the Tisdale score (Fig 1a). Simultaneously, a decrease from 67 (30.0%) to 51 (24.0%) of serious cardiovascular ADE was noticed, i.e., QT interval prolongation (before: n = 37, 17.0%; after: n = 30 14.0%), Torsades de Pointes (before: n = 21, 9.0%; after: n = 15, 7.0%) and SS (before: n = 9, 4.0%; after: n = 6, 3.0%).

A significant reduction in the Tisdale score was observed after the hypothetical clinical management of drugs in certain groups of patients (Supporting Information, S1 Table). Furthermore, statistical analysis was performed to verify other characteristics, in addition to those reflected in the Tisdale score, that may have contributed to the high cardiovascular risk values (Table 2).

**Table 2:** Variation in the prevalence ratio between the Tisdale scores related to QT interval prolongation in patients diagnosed with COVID-19 admitted to the ICU of a Brasilia reference hospital from March to December 2020. Systemic arterial hypertension (SAH); prevalence ratio (PR); 95% confidence interval (95%CI).

Features	*Score increased*		Univariate model	
	n	%	PR	95%CI
***Gender***				
Male	19	27.5%	1.455	0.64 - 3.73
Female	7	18.9%	1.000	–
***Race***				
White	5	38.5%	1.000	–
Multiracial	7	25.9%	0.674	0.21-2.28
***Health professional***				
Yes	1	33.3%	1.000	–
No	24	24.2%	0.727	0.15-13.00
***SAH***				
Yes	15	24.6%	1.000	–
No	11	24.4%	0.994	0.44 - 2.15
***Elderly***				
Yes	16	28.1%	1.000	–
No	10	20.4%	0.727	0.32 - 1.58
***Obesity***				
Yes	14	31.8%	1.000	–
No	12	19.4%	0.608	0.28 - 1.32
***Smoker***				
Yes	10	29.4%	1.000	–
No	16	22.2%	0.756	0.35 - 1.72

Clinical data showed that men have a 1.5 times greater risk of having an increased Tisdale score. In addition, patients without SAH have a 99% lower chance of experiencing an increased Tisdale score. When it comes to age, individuals under 60 are at 72.7% lower risk of increasing their Tisdale score than older people. The risk of having an increased Tisdale score is 60.8% lower for eutrophic patients compared to obese patients. White individuals are less likely to have an increased QT interval score, at a 67.4% lower risk. Healthcare professionals have a 72.7% lower chance of experiencing an increase in QT interval score. Lastly, regarding the health condition of dependence, smokers have a 75.6% lower risk of having an increased Tisdale score compared to those who do not use tobacco (Table 2).

Finally, all medications that cause serious cardiovascular ADE were grouped by therapeutic class and frequency of use. The most used drugs were fentanyl (n = 1993; 4.8%), ondansetron (n = 1362; 3.3%), dexmedetomidine (n = 731, 1.8%), and azithromycin (n = 506, 1.3%) (Supporting Information, S2 Table).

## 4. Discussion

The assessment of demographic data is relevant to this study, especially given the pandemic context in which it was conducted. The average ICU stay was 21 days. This result is similar to other published studies regarding the same period of COVID-19 pre-vaccination. In fact, a systematic review and meta-analysis conducted by Alimohamadi et al. found that ICU stays for patients with COVID-19 in South America were approximately 20 days [31]. The high hospitalization costs during this period had a significant impact on health and varied considerably across countries. For example, the cost of hospitalization per person in Hungary reached €30,990 [32], while in France, it was €1,425 [33]. The daily cost per person in the United States was $4,300 [34], whereas in Brazil, the average daily cost was $2,000 [35].

Among the comorbidities identified in this study, the most prevalent was SAH (n = 61; 58%), followed by Diabetes mellitus (n = 38; 36%). The review conducted by Salabei et al. identified that pre-existing SAH is directly related to increased mortality and severity of COVID-19 [12], predisposing to more serious ADE [12,36]. Another review showed that approximately a quarter of patients with COVID-19 had a cardiac injury, even without pre-existing cardiovascular disease [37], corroborating the high prevalence of hypertension in the findings of the present study.

Furthermore, a 207-study systematic review found that mortality increased by 6% in patients with SAH, and the chance of progression to severe COVID-19 was 12.1% higher. This study also showed that the probability of a patient with Diabetes mellitus having a worsening of COVID-19 is 13.2%, with a 5.6% increased risk of death [5]. Another review demonstrated that patients with Diabetes mellitus and elevated blood glucose levels produce more pro-inflammatory cytokines, increasing the susceptibility and severity of infections by weakening the immune system. Furthermore, there is clinical evidence that hyperglycemia also increases the expression of ACE2 in the lung and other tissues [38].

The present study showed a male prevalence of 65% (n = 69) for ICU patients with COVID-19. A review conducted by Zhang et al. showed that males have a higher infection risk than females. The cause may be related to hormonal effects during inflammation since testosterone acts as a protease catalyst, increasing the level of ACE2 cell receptors and TMPRSS2 molecules, which facilitates the entry of the virus into cells [39]. In addition, a systematic review demonstrated that men have a 5% higher chance of developing severe COVID-19 [5].

In this study, the mean age of patients was 60 years old, with 55% (n = 59) being over 60 years old. According to the literature, this group’s chance of developing severe COVID-19 increases by 6.6% [5]. Indeed, advanced age is related to greater susceptibility to adverse drug reactions and infections, especially respiratory diseases, in addition to higher levels of pro-inflammatory cytokines and a higher risk of hospitalizations with poor prognosis and death [5].

Another striking feature in this study is the occurrence of obesity in 39.6% (n = 42) of patients. In the systematic review by Izcovich et al., obesity increases the chance of developing severe COVID-19 by 16.7% and the chance of death by 3.1% due to the increased risk of developing respiratory distress syndrome [5,36].

Another aspect analyzed was the use of medications for the treatment of chronic diseases during the pandemic, particularly medicines for mental disorders, which accounted for 24% of patients (n = 26). The literature shows that during the COVID-19 pandemic, an increase in the prescription of antidepressants associated with typical and atypical antipsychotics occurred. A systematic review analyzed the increase in the consumption of psychotropic drugs on a global scale for the treatment of psychosomatic diseases during the COVID-19 pandemic, demonstrating a considerable rise in depression and anxiety [40].

Among patients with diseases affecting the central nervous system, two patients were identified with schizophrenia who chronically used risperidone (atypical antipsychotic), levomepromazine, and haloperidol (typical antipsychotic). Antipsychotics in the ICU are used to treat delirium, which is associated with increased length of hospital stay and mortality [41]. In fact, such patients had a higher risk of synergism of serious cardiovascular ADE. Although the therapeutic class of typical and atypical antipsychotics is related to serious cardiovascular ADE, the clinical need prevails over cardiovascular risk. The UK guideline analyzed the cost-effectiveness of antipsychotics within the ICU, demonstrating probable benefit [42].

Another relevant aspect was the self-medication for the treatment of COVID-19. Kazemioula et al. showed a prevalence of self-medication in the general population of 48%, with 41% in patients with COVID-19 [43]. In addition, it also identified an increase in the prevalence of self-medication in 2021 compared to the previous year [43]. The difficulty in accessing health care during the pandemic period increased self-medication. The present study shows 21% (n = 20) of self-medication. However, the result may be underestimated, as many patients during this period were hesitant to disclose this information due to the politicization of medication use in Brazil [44].

The time frame selected for this study, from March to December 2020, coincides with the pre-vaccine period of the COVID-19 pandemic. The overcrowding of ICUs during this interval seems to be an ideal scenario to assess the potential for intervention in medical prescriptions, suggesting a reduction in cardiovascular risk through the optimized use of medications. In particular, this assessment is even more relevant considering that the high death rate during this period may be, to some extent, related to cardiovascular issues.

In this sense, the central focus of this study was to determine the cardiovascular risk analysis using the Tisdale score in patients with COVID-19 admitted to the ICU, as well as to assess the impact that the simple replacement/suspension of treatment with medications with the same clinical indication, but with less potential for cardiovascular damage, could bring, carried out with the help of the MedUTI software to guide the replacements.

A significant reduction in serious ADE, including a considerable decrease in the high-risk classification of around 53%, was observed. Indeed, the simulation of hypothetical intervention in prescriptions with moderate and high risk is recommended in clinical practice, together with monitoring of patients by echocardiogram after five half-lives of drugs that present ADE of QT interval prolongation [23].

The study showed that 30% (n = 67) of the medications used by patients presented a risk of serious cardiovascular ADE and synergism of serious cardiovascular ADE. This percentage is considered high, especially for patients admitted to the ICU, but statistics on this subject are scarce. In fact, in a recent literature review, the relationship between cardiac arrhythmia and ICU patients indicated the need to manage the medications used. Patients are susceptible to drug-drug interactions, and medications that cause proarrhythmic effects or that alter metabolism and inhibit CYP are contraindicated, as they can exacerbate the risk of arrhythmias, causing QT interval prolongation, Torsades de pointes, and SS [45,46].

The therapeutic classes with a recommendation for replacement or suspension include opioid narcotic analgesics, antidepressants, typical antipsychotics, antiemetics/antinauseants, antimalarials, anti-HIV antivirals, and muscle relaxants.

Methadone and tramadol cause QT interval prolongation, Torsades de pointes, and SS, and are used to reduce pain [24]. Despite their widespread use in the COVID-19 scenario, considering the pain scale, they should be replaced with morphine, fentanyl, and hydromorphone to reduce cardiovascular risk [24]. There is ample literature to support this replacement. In fact, a review on analgesia in the MEDLINE and EMBASE databases showed that the most prescribed opioid is morphine, followed by fentanyl. Furthermore, an interview with 273 intensive healthcare professionals confirmed the preference for morphine and fentanyl as opioid analgesics used in the ICU [26]. Another study analyzing the opioid analgesics prescribed in the ICUs of 269 hospitals showed that the most prescribed were also morphine, followed by fentanyl [25]. Finally, another comprehensive review described that the opioid analgesics historically most prescribed in the ICU include morphine, hydromorphone, and fentanyl, preferably [25,47].

The sedatives respond to a crucial pharmacological group used in the ICU in COVID-19 cases [27]. The proposed drug management suggests replacing typical antipsychotics and tricyclic antidepressants for sedation with propofol, benzodiazepines, dexmedetomidine, or ketamine. In fact, there was no standardized sedation protocol for patients with COVID-19 [27], underscoring that the proposed substitutions would provide greater cardiovascular safety without compromising the required sedation.

The analysis also revealed frequent therapeutic substitutions among sedative and antipsychotic agents during the study period [48]. During the COVID-19 pandemic, drug shortages and the urgent need for rapid sedation often led to substitutions that reflected operational constraints rather than optimal clinical choices. In the MedUTI database, these substitutions were recorded as part of real-world practice rather than as recommended therapeutic strategies.

In patients with hyperactive delirium, where antipsychotics are considered first-line, substitution with benzodiazepines, propofol, or dexmedetomidine occurred in some cases due to unavailability or contraindication of standard agents. However, such substitutions may not be clinically appropriate. For instance, benzodiazepines can worsen delirium, propofol may cause excessive sedation in non-ventilated patients, and dexmedetomidine carries cardiovascular risks such as bradycardia and hypotension. These findings highlight the need for continuous pharmacovigilance and reinforce the importance of clinical decision-support tools such as MedUTI in guiding rational and safer pharmacotherapy in the ICU, particularly in scenarios of high demand and limited resources [48].

Another therapeutic class commonly used in ICUs is antiemetics/antinauseants. Among the prescriptions analyzed in this study, replacing ondansetron and domperidone with metoclopramide is highly recommended. The prescription of an antiemetic aims to increase the gastrointestinal motility of patients admitted to the ICU, as well as to prevent ADE of nausea and vomiting caused by other medications with emetogenic potential [28]. In this sense, metoclopramide would perfectly meet this need without the cardiovascular drawbacks discussed. Furthermore, a systematic review and meta-analysis with randomized data showed moderate evidence for the prescription of prokinetics; however, without probable benefit in reducing food intolerance in critically ill patients and decreasing pneumonia and mortality [28].

Finally, the impact on cardiovascular risk was also assessed when drugs for the treatment of COVID-19 without clinical evidence, which presented serious ADE with the potential to increase QT interval prolongation, were discontinued.

The results obtained in a systematic review and network meta-analysis identified the drugs used to treat COVID-19 during the pandemic. The therapeutic classes of systemic corticosteroids, interleukin-6 receptor antagonists, and Janus kinase inhibitors were the only ones that presented clinical evidence of reducing mortality in hospitalized patients [29]. These findings are corroborated by extensive scientific literature, including the systematic review by Wang et al., which concluded that no clinical trial confirmed the effectiveness of the drugs tested to date for the treatment of COVID-19 [30]. Medications used in the hospital studied without clinical evidence for treating COVID-19 include antimalarials (hydroxychloroquine and chloroquine), anti-HIV antivirals (lopinavir and ritonavir), and muscle relaxants (cyclobenzaprine).

In particular, antimalarials cause serious ADE of QT interval prolongation, which may have increased the risk of death in patients who used such medications. In fact, the systematic review and meta-analysis conducted by Thoguluva Chandrasekar et al. al, showed that patients who used hydroxychloroquine as a form of treatment for COVID-19 had significantly higher mortality (odds ratios: 1.36; 95% confidence interval: 0.97–1.89) and had more occurrences of ADE (odds ratios: 3.88; 95% confidence interval: 1.60–9.45) [49].

Although QT-interval prolongation served as a crucial measurable endpoint in this study, it represents only one dimension of cardiovascular safety in critically ill patients. The analyses from the MedUTI platform revealed a broader, multifactorial landscape of pharmacotherapeutic risk in the ICU. Throughout the COVID-19 pandemic, clinicians faced simultaneous challenges, including drug shortages, rapidly escalating sedation needs, management of hyperactive delirium, hemodynamic instability, and fluctuating organ dysfunction [48]. These overlapping pressures shaped prescribing patterns and, at times, contributed to potentially unsafe therapeutic substitutions.

Thus, cardiovascular outcomes, such as QT prolongation, Torsades de pointes, or SS, must be interpreted into this broader context of critical-care pharmacology [50]. By integrating daily prescription data with computerized drug-risk mapping, the present study highlights the importance of comprehensive pharmacovigilance and supports the development of intelligent decision-support systems, such as MedUTI, to guide safer and evidence-based medication management in high-acuity environments [51].

## 5. Research limitations

The literature demonstrates the usefulness of the Tisdale score for assessing patients’ cardiovascular risk [52]. However, this instrument was designed specifically for the cardiovascular ICU, and some parameters do not fit perfectly in a regular ICU. Furthermore, the special conditions of COVID-19 patients during the pandemic period make clear the need to adapt this tool [23,52].

In fact, a retrospective study that applied the Tisdale score to patients with COVID-19 admitted to Ascension St. John Hospital in Detroit, USA, showed that only 11.8% of patients scored 7 or higher, revealing a medium risk [52]. These results suggest an underestimation of cardiovascular risk. From the data analyzed in the present study, it is possible to infer the need to include other variables in the Tisdale score. In fact, it was possible to verify that the etiologies predisposing to QT interval prolongation in these patients are multifactorial, with medications appearing to collaborate in the process [52].

In contrast, the MedUTI software used as a complementary method for assessing cardiovascular risk in prescriptions was created during the pandemic so that it could be tested for the pathophysiology of COVID-19, and appears to have helped correct this distortion. Furthermore, the Tisdale score assigns an additional score to female patients. Contradictorily, the survey presented here showed that cardiovascular risk is higher in men. This result may be influenced by the particular clinical conditions of COVID-19, which has a higher incidence in males, in addition to biological and sociocultural characteristics [3,39,53].

Another statistical bias is in the clinical outcome of the patients since 73.58% (n = 78) of the patients analyzed died. This is undoubtedly a justifiable fact, given the period analyzed before vaccination. Finally, another limitation when analyzing and discussing the data is the absence of patients’ electrocardiograms. During the pandemic, the exams were performed at the patient’s bedside and were not added to the electronic record.

Finally, improving the Tisdale score also requires prospective studies conducted under more appropriate conditions and controlled environments, with access to periodic examinations.

## 6. Conclusion

Medication management considering cardiovascular risk, combined with the support of health technologies, has demonstrated great potential to assist healthcare professionals in making rapid decisions that can prevent ADE risks for ICU patients. In particular, the chaotic scenario of ICU overcrowding in the pre-vaccination period of the COVID-19 pandemic highlights the importance of more accurate decision-making regarding patient medication prescriptions. Indeed, an impressive 96% of prescriptions presented a high cardiovascular risk, and not by chance, 74% of patients died during this period. In fact, serious ADE such as QT interval prolongation, Torsades de pointes, and SS, as well as the synergism of these ADE, may have contributed significantly to this unfavorable clinical outcome. The hypothetical intervention simulated in these prescriptions promoted a significant reduction in cardiovascular risk by more than half, demonstrating that the use of technological tools in health can be a powerful medical support tools with an impact on the length of hospital stay, health system expenses, and, above all, on patient survival.

## Supporting information

S1 Table Mean, standard deviation, and multifactorial ANOVA of the Tisdale scores before and after the hypothetical clinical management of drugs in patients diagnosed with COVID-19 admitted to the ICU of a Brasilia reference hospital from March to December 2020. Systemic arterial hypertension (SAH).
(DOCX)

S2 Table Therapeutic classification of drugs with potential for serious cardiovascular ADE. (DOCX)

S1 Dataset Anonymized participant information collected is available at https://doi.org/10.34740/kaggle/dsv/11795374. (DOCX)
