# Supplementary material for: Risk assessment for cardiovascular adverse drug events in the ICU: Case study on COVID-19 patients
Source: PLoS One. 2026 Mar 24;21(3):e0345280. doi: 10.1371/journal.pone.0345280 (PMC13012493; doi:10.1371/journal.pone.0345280)
Supplement: S1 Table — Systemic arterial hypertension (SAH). (DOCX) [file pone.0345280.s001.docx]

**S1 Table.** Mean, standard deviation, and multifactorial ANOVA of the Tisdale scores before and after the hypothetical clinical management of drugs in patients diagnosed with COVID-19 admitted to the ICU of a Brasilia reference hospital from March to December 2020. Systemic arterial hypertension (SAH).

| **Features** | **QT interval before** | **QT interval after** | **p-value** |
| --- | --- | --- | --- |
| ***Gender*** |  |  |  |
| Male | 9.43 (1.25) | 8.61 (1.87) | <0.001 |
| Female | 10.65 (1.65) | 10.08 (2.06) |  |
| ***Comorbidity*** |  |  |  |
| None | 9.18 (0.50) | 8.09 (1.51) | <0.001 |
| one | 9.76 (1.23) | 9.06 (1.94) |  |
| two | 9.54 (1.44) | 8.59 (2.09) |  |
| Three or more | 10.75 (1.99) | 10.43 (1.95) |  |
| ***Diabetes mellitus*** |  |  |  |
| Yes | 10.18 (1.77) | 9.71 (2.05) | 0.006 |
| No | 9.68 (1.33) | 8.79 (2.00) |  |
| ***SAH*** |  |  |  |
| Yes | 10.16 (1.68) | 9.43 (2.20) | 0.004 |
| No | 9.44 (1.16) | 8.71 (1.78) |  |
