# Supplementary material for: Risk assessment for cardiovascular adverse drug events in the ICU: Case study on COVID-19 patients
Source: PLoS One. 2026 Mar 24;21(3):e0345280. doi: 10.1371/journal.pone.0345280 (PMC13012493; doi:10.1371/journal.pone.0345280)
Supplement: S2 Table — (DOCX) [file pone.0345280.s002.docx]

**S2 Table.** Therapeutic classification of drugs with potential for serious cardiovascular ADE.

| **ADE** | **Medication** | **Therapeutic class** | **n** | **%** |
| --- | --- | --- | --- | --- |
|  |  |  | **6.149** | **100.00** |
| QT interval prolongation | Ondansetron  Domperidone | Antiemetics/antinauseants | 1395 | 22.69 |
|  | Dexmedetomidine | Sedative | 731 | 11.90 |
|  | Haloperidol  Chlorpromazine  Levomepromazine  Quetiapine  Risperidone | Antipsychotics | 402 | 6.53 |
|  | Methadone  Tramadol | Narcotic analgesics | 315 | 5.1 |
|  | Amiodarone | Antiarrhythmic | 268 | 4.36 |
|  | Ciprofloxacin  Gentamicin  Sulfamethoxazole + trimethoprim  Levofloxacin  Moxifloxacin | Antibiotic | 153 | 2.50 |
|  | Amitriptyline  Sertraline  Fluoxetine  Escitalopram  Citalopram  Fluvoxamine  Nortriptyline | Antidepressants | 109 | 1.80 |
|  | Chloroquine  Hydroxychloroquine | Antimaláricos | 79 | 1.30 |
|  | Anidulafungin  Ketoconazole  Fluconazole | Antifungals | 49 | 0.80 |
|  | Promethazine | Antihistamine | 21 | 0.34 |
|  | Propranolol  Clonidine | Antihypertensive | 18 | 0.30 |
|  | Lopinavir + Ritonavir | Antiviral Anti-HIV | 13 | 0.21 |
|  | Atropine | Anticholinergic | 8 | 0.13 |
|  | Umeclidinium + Vilanterol | Anti-asthmatic | 6 | 0.10 |
|  | Tacrolimus | Immunosuppressant | 5 | 0.08 |
|  | Cyclobenzaprine | Muscle relaxant | 2 | 0.03 |
| Torsades de pointes | Ketoconazole  Fluconazole | Antifungals | 2499 | 40.64 |
|  | Dexmedetomidine | Sedative | 731 | 11.90 |
|  | Azithromycin  Levofloxacin  Moxifloxacin  Ciprofloxacin  Sulfamethoxazole + trimethoprim | Antibiotic | 641 | 10.42 |
|  | Amiodarone | Antiarrhythmics | 268 | 4.36 |
|  | Methadone | Narcotic analgesic | 237 | 3.90 |
|  | Haloperidol  Levomepromazine | Antipsychotics | 83 | 1.35 |
|  | Citalopram  Sertraline  Fluvoxamine | Antidepressant | 21 | 0.34 |
|  | Lopinavir + Ritonavir | Antiviral Anti-HIV | 13 | 0.21 |
|  | Ondansetron | Antiemetics/antinauseants | 10 | 0.16 |
| Serotonin syndrome | Fentanyl | Sedative | 1993 | 32.41 |
|  | Ondansetron | Antiemetics/antinauseants | 1395 | 22.70 |
|  | Tramadol | Narcotic analgesic | 78 | 1.27 |
|  | Citalopram  Fluoxetine  Escitalopram  Sertraline  Fluvoxamine | Antidepressant | 57 | 0.92 |
|  | Indapamide | Antihypertensive | 39 | 0.63 |
|  | Chloroquine  Hydroxychloroquine | Antimalarials | 33 | 0.40 |
|  | Cyclobenzaprine | Muscle relaxant | 9 | 0.03 |
