# Supplementary material for: Risk assessment for cardiovascular adverse drug events in the ICU: Case study on COVID-19 patients
Source: PLoS One. 2026 Mar 24;21(3):e0345280. doi: 10.1371/journal.pone.0345280 (PMC13012493; doi:10.1371/journal.pone.0345280)
Supplement: S1 Dataset — (DOCX) [file pone.0345280.s003.docx]

**S3 Dataset**. Anonymized participant information collected is available at https://doi.org/10.34740/kaggle/dsv/11795374
